# The Obesity-Related Peptide Leptin Sensitizes Cardiac Mitochondria to Calcium-Induced Permeability Transition Pore Opening and Apoptosis

**DOI:** 10.1371/journal.pone.0041612

**Published:** 2012-07-25

**Authors:** Eduardo Martinez-Abundis, Venkatesh Rajapurohitam, James V. Haist, Xiaohong T. Gan, Morris Karmazyn

**Affiliations:** Department of Physiology and Pharmacology, Schulich School of Medicine and Dentistry, University of Western Ontario, London, Ontario, Canada; Temple University, United States of America

## Abstract

The obesity-related 16 kDa peptide leptin is synthesized primarily in white adipocytes although its production has been reported in other tissues including the heart. There is emerging evidence that leptin may contribute to cardiac pathology especially that related to myocardial remodelling and heart failure. In view of the importance of mitochondria to these processes, the goal of the present study is to determine the effect of leptin on mitochondria permeability transition pore opening and the potential consequence in terms of development of apoptosis. Experiments were performed using neonatal rat ventricular myocytes exposed to 3.1 nM (50 ng/ml) leptin for 24 hours. Mitochondrial transition pore opening was analyzed as the capacity of mitochondria to retain the dye calcein-AM in presence of 200 µM CaCl2. Leptin significantly increased pore opening although the effect was markedly more pronounced in digitonin-permeabilized myocytes in the presence of calcium with both effects prevented by the transition pore inhibitor sanglifehrin A. These effects were associated with increased apoptosis as evidenced by increased TUNEL staining and caspase 3 activity, both of which were prevented by the transition pore inhibitor sanglifehrin A. Leptin enhanced Stat3 activation whereas a Stat 3 inhibitor peptide prevented leptin-induced mitochondrial transition pore opening as well as the hypertrophic and pro-apoptotic effects of the peptide. Inhibition of the RhoA/ROCK pathway prevented the hypertrophic response to leptin but had no effect on increased pore opening following leptin administration. We conclude that leptin can enhance calcium-mediated, Stat3-dependent pro-apoptotic effects as a result of increased mitochondrial transition pore opening and independently of its hypertrophic actions. Leptin may therefore contribute to mitochondrial dysfunction and the development of apoptosis in the diseased myocardium particularly under conditions of excessive intracellular calcium accumulation.

## Introduction

Leptin, the product of *ob/ob* gene is an important adipocyte-derived satiety factor whose plasma levels are profoundly increased in obesity in direct proportion to the degree of adiposity [1]. A number of studies have shown that plasma levels of the 16 kDa peptide are elevated in patients with heart failure [2]–[4] whereas others have failed to show an increase [5]. In addition to adipocytes as a source of leptin production the peptide is also produced by the heart and may serve to regulate cardiac function in a paracrine or autocrine manner [6], [7]. Leptin exerts a myriad of effects through its cell surface receptors termed ObR or LepR, of which there are six isoforms which belong to the class 1 cytokine receptor family [8]. Interestingly, these receptors are upregulated in the human failing myocardium [9]. The nature of leptin’s effects on the heart, especially pertaining to its possible role in cardiac pathology is not completely understood. There is extensive evidence that leptin induces cardiomyocyte hypertrophy under both *in vivo* and *in vitro* conditions and leptin receptor inhibition ameliorates the myocardial response to chronic coronary artery ligation [10]–[15]. Leptin has recently been shown to increase myocardial collagen levels concomitant with diastolic dysfunction when administered to obese *ob/ob* mice [16]. Others however have shown a salutary role of leptin receptor deletion in the postinfarcted heart [17] although, as recently discussed [18], the bases for such differences are not well understood.

A major pathway for leptin’s actions is *via* JAK2/STAT3 activation [19] although we have also demonstrated that leptin activates the Ras homolog gene family, member A/Rho-associated, coiled-coil containing protein kinase (RhoA/ROCK) pathway which in turn plays a critical role in mediating leptin’s hypertrophic effect [12]. A particularly important process in the progression of hypertrophy to heart failure is related to mitochondrial remodelling which could contribute to cardiac pathology through the activation of apoptosis especially by enhancing transition to heart failure [20], [21]. Mitochondria are considered important regulators of apoptosis by virtue of their ability to release pro-apoptotic factors following appropriate stimulation resulting in DNA cleavage and caspase activation [22]–[24]. Different mechanisms have been described that can trigger or facilitate mitochondria-mediated apoptosis including the imbalance between pro- and anti-apoptotic Bcl-2 family members (Bax and Bcl-2 respectively), increased oxidative stress resulting in mitochondrial permeability transition pore (mPTP) opening as well as changes in the balance between mitochondrial fission and fusion proteins [22]–[25]. Currently, there is no information as to whether exposure to leptin can modify cardiac mitochondrial function. Accordingly, the present study was performed to determine the effects of 24 hour exposure to leptin on mitochondrial function and possible relevance to the apoptotic process. This was done using cultured ventricular myocytes in which we determined the effects of 24 hour exposure to leptin or in myocytes pretreated with leptin for 24 hours followed by exposure to calcium overload as a result of membrane permeabilization with digitonin.

**Figure 1:**
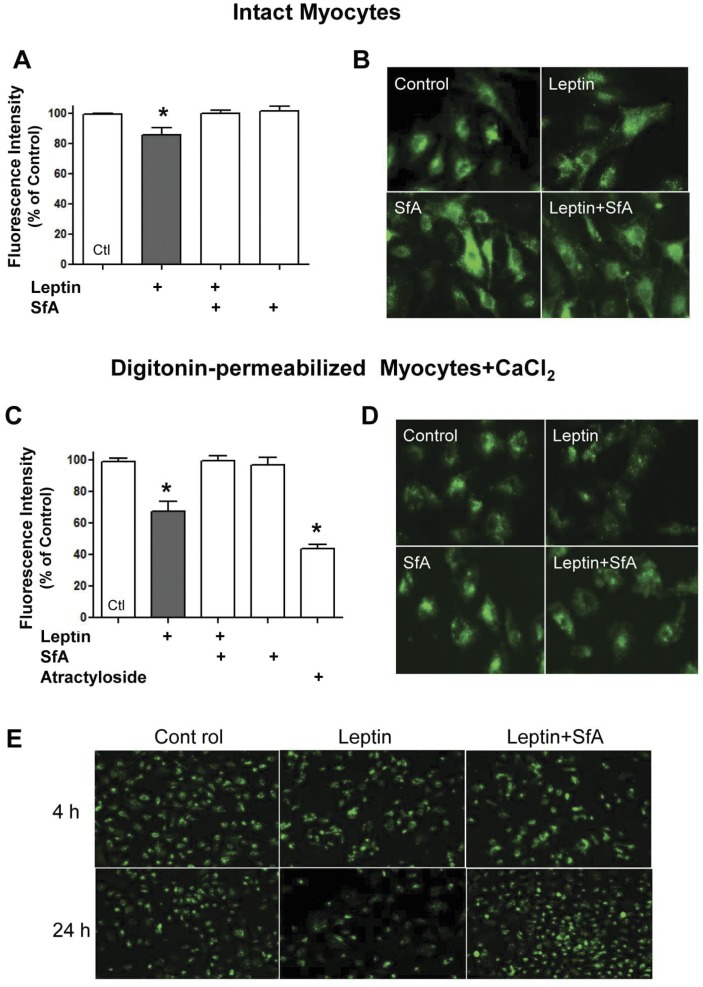
Effect of leptin on mPTP opening. Panels A and B show quantified data and examples of calcein fluorescence, respectively in intact myocytes treated for 24 hours under different experimental conditions in the absence or presence of leptin. Panels C and D show identical experiments for digitonin-permeabilized myocytes in the presence of 200 µM CaCl2. The effect of atractyloside is shown for comparison purposes. Panel E shows representative fluorescence images for myocytes treated for either 4 or 24 hours and demonstrates no effect on mPTP opening after 4 hours of leptin treatment. Leptin-induced mPTP opening, depicted as a reduction in calcein fluorescence, was prevented by sanglifehrin A (SfA). For panels A and C, values indicate mean + SEM, N = 8. *P<0.05 from all groups. Images in panels B and D were obtained using 400X magnification whereas images in panel E were obtained using a magnification of 200X.

## Methods

### Ethics Statement

All protocols for the use of animals are in accordance with the University of Western Ontario animal care guidelines. These protocols conform to the guidelines of the Canadian Council on Animal Care (Ottawa, ON, Canada) and have been approved by the University of Western Ontario Council on Animal Care (protocol number 2009–020).

**Figure 2:**
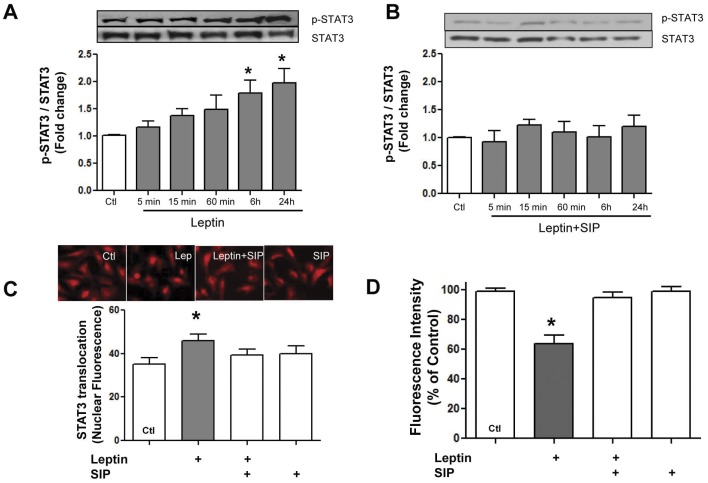
Evidence for Stat3 involvement in leptin-induced increase in mPTP opening. Panels A and B show time-dependent leptin-induced increase in Stat3 phosphorylation in the absence or presence of the Stat inhibitory peptide (SIP), respectively. Panel C demonstrates fluorescent images and corresponding quantified data indicating increased Stat3 translocation into nuclei whereas panel D shows prevention of leptin-induced mPTP opening by SIP. Values indicate mean + SEM, N = 8. *P<0.05 from control (Ctl).

### Primary Culture of Neonatal Cardiomyocytes

To prepare primary cultures of cardiomyocytes 1–3 days old Sprague-Dawley rats were sacrificed by decapitation, the hearts were removed and minced with scissors and then subjected to seven serial digestions with collagenase. Cell suspensions were pooled and cells were recovered by centrifugation for 5 minutes (500 g at 4°C), re-suspended in culture medium (DMEM F12 containing 10% of fetal bovine serum, FBS) and plated in Primaria cell culture dishes after incubation for 2 hours at 37°C with 5% CO2 to eliminate non-cardiomyocytes. After 48 hours incubation, cells were maintained for 24 hours in FBS-free media. Cardiomyocytes were treated with vehicle (control) or 3.1 nM leptin (50 ng/ml, Sigma-Aldrich, Oakville, Ontario, Canada) for 24 hours after 30 minutes of incubation in the absence or presence of the STAT3 inhibitor peptide (SIP, 1 mM, Calbiochem, San Diego, CA, USA); the mPTP inhibitor Sanglifehrin A (0.2 µM, gift of Novartis Pharmaceuticals, Basel, Switzerland) or the ROCK inhibitor Y27632 (10 µM, Sigma-Aldrich). For some experiments we also determined the effect of the Stat1 inhibitor fludarabine (9-β-D-arabinofuranosyl-2-fluoroadenine 5′-phosphate, 50 µM, Sigma-Aldrich) which was also added 30 minutes before leptin administration. The concentrations of Stat inhibitors used in the present study reflected their ability to completely prevent leptin-induced increased Stat phosphorylation. Thus, for SIP, a 1 mM concentration reduced Stat3 phosphorylation by 100% whereas SIP concentrations of 10 µM, 100 µM and 500 µM reduced peak Stat3 phosphorylation 24 hours after leptin addition by 37±4%, 69±11% and 87±7% respectively (N = 3). Similarly, a fludarabine concentration of 50 µM completely suppressed peak leptin-induced Stat1 phosphorylation at 15 minutes whereas with fludarabine concentrations of 1 µM, 5 µM and 25 µM Stat1 phosphorylation at this time point was decreased by 17±2%, 37±9% and 63±12%, respectively (N = 3).

**Figure 3:**
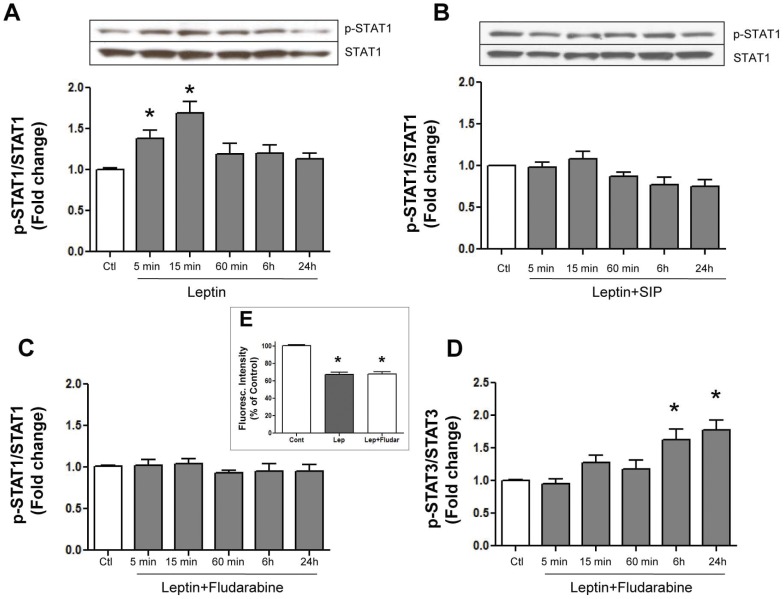
The effect of SIP and fludarabine on leptin-induced Stat1 and Stat3 activation. Panels A and B show time-dependent leptin-induced increases in Stat1 phosphorylation in the absence or presence of the Stat inhibitory peptide (SIP), respectively. Panel C shows complete inhibition of leptin-induced Stat1 phosphorylation by fludarabine although the increased Stat3 phosphorylation was unaffected (panel D). As shown in the inset (panel E) fludarabine had no effect on leptin-induced mPTP opening. Values indicate mean + SEM, N = 5. *P<0.05 from control (Ctl).

### Myocyte Surface Area Analysis

After treatments, images were obtained from cardiomyocytes using a Leica inverted microscope equipped with a Polaroid digital camera at 200X magnification. The myocyte surface area was measured using SigmaScan Pro 5 software (SPSS Inc., Chicago IL, USA) and expressed as fold changes with respect to the control.

### RNA Isolation and Reverse Transcription

Total RNA was extracted using TRIZOL (Invitrogen, Carlsbad, CA, USA) following the manufacturer’s instructions. RNA (4 µg) was used to synthesize the first strand of cDNA using M-MLV-Reverse Transcriptase (Invitrogen) and was used afterwards as a template in the PCR reactions.

**Figure 4:**
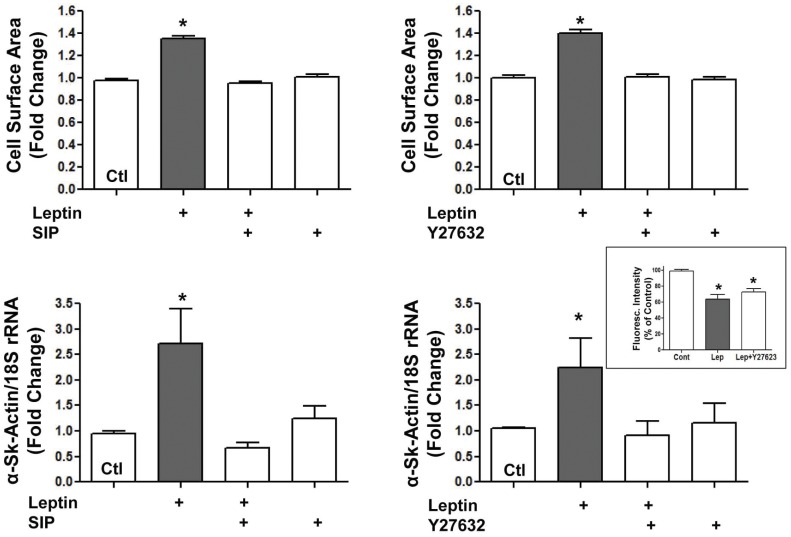
Inhibition of leptin-induced hypertrophy as assessed by cell surface area and α-skeletal actin expression by the Stat inhibitory peptide (SIP) and the ROCK inhibitor Y27632. Inset shows a lack of effect of Y27632 on leptin-induced mPTP opening. Values indicate mean + SEM, N = 8. *P<0.05 from control (Ctl).

### Real-Time PCR

The expression of α-skeletal actin and 18S rRNA genes was determined in a 10 µl reaction volume using SYBR Green Jumpstart Taq ReadyMix DNA polymerase (Applied Biological Materials Inc., Richmond, BC, Canada), and fluorescence was measured and quantified using the DNA Engine Opticon 2 System (Bio-Rad Laboratories, Hercules, CA, USA). Amplification was performed using the following primers: 5′-CACGGCATTATCACCAACTG-3′ (forward) and 5′-CCGGAGGCATAGAGAGACA-G-3′ (reverse) for α-skeletal actin, and 5′-GTAACCCGTTGAACCCCATT-3′ (forward) and 5′-CCATCCAATCGGTAGTAGCG-3′ (reverse) for 18S rRNA. PCR conditions to amplify the genes were 30 seconds at 94°C followed by annealing at 54°C for 20 seconds for 18S rRNA and 60°C for 25 s for α-skeletal actin, with further elongation at 72°C for 30 seconds. α-skeletal actin was amplified for 40 cycles, whereas 18S rRNA was amplified for 35 cycles. 18S rRNA gene expression was used as a control.

### Mitochondrial Permeability Transition Pore Opening

To measure the mPTP in intact cardiomyocytes cells were plated in 24 well plates (0.5×10^6^ cells/well), loaded with calcein-acetoxymethylester 1 mM (calcein-AM, Molecular Probes, Eugene, OR, USA) in presence of 5 mM of cobalt chloride to quench the cytosolic and nuclear fluorescence for 30 minutes at 37°C. Fluorescence was measured in a SpectraMax M5 microplate reader (Molecular Devices, Sunnyvale, CA, USA) at 494 nm excitation and 517 nm emission. Alternatively, to measure the mPTP opening *in situ*, cells were washed twice with PBS after loading with calcein-AM in presence of cobalt chloride and permeabilized with digitonin (10 µM) for 10 minutes in a cytosolic buffer (KCl 100 mM, KH2PO4 1 mM, MgCl2 1 mM, EGTA 50 µM, HEPES 10 mM, pH 7.3) containing succinate (5 mM) as oxidizable substrate and 1 mg/ml rotenone. After washing with the same buffer lacking digitonin, cells were exposed to 200 µM CaCl2 and images were captured with a fluorescence microscope (Olympus AX70 Research Microscope). Fluorescence intensity of individual cells was measured using SigmaScan Pro 5 software.

### Western Blotting

Cell were washed twice with PBS and harvested in lysis buffer containing Tris-base (50 mM), NaCl (150 mM), Triton-X100 (1%), glycerol (10%), EDTA (2 mM), EGTA (2 mM), NaF (50 mM), Na2PO4 (10 mM), β-glycerophosphate (40 mM), Na3VO4 (200 µM) after which myocytes were homogenized by ten passages through a syringe needle. The total homogenate was clarified by centrifugation for 10 minutes at 10,000 g at 4°C and the supernatant was mixed with 6X sample buffer (Tris-HCl 375 mM, SDS 9%, glycerol 50%, β-mercaptoethanol 9% and bromophenol blue 0.03%) after protein quantification. Proteins were resolved in an acrylamide-bis acrylamide 12% gel, transferred to 0.45 µm nitrocellulose membranes and, after blocking with 5% skim dry milk, were incubated overnight at 4°C with the primary antibodies (rabbit anti-Bax, rabbit anti-cytochrome c, mouse anti-Bcl2, mouse anti-actin) diluted 1∶1000 with 3% BSA in Tris-buffered saline with Tween-20 followed by 2 hour incubation with horseradish peroxidase-conjugated secondary antibodies at room temperature. Proteins were detected with ECL Western blotting detection reagents.

### TUNEL Assay

The terminal deoxynucleotidyl transferase dUTP nick end labeling (TUNEL) method that detects DNA fragmentation was carried out as a marker of apoptosis in cardiomyocytes previously fixed for 30 minutes with 4% paraformaldehyde, permeabilized for 15 minutes with 0.2% of Triton-X100 and blocked with 1% bovine serum albumin. Detection was carried out using the manufacturer’s instructions (TUNEL Apoptosis Detection Kit; Millipore, Temecula, CA, USA).

### Caspase 3 Activity

Activity of caspase 3 was quantified in cell homogenates with a commercial kit according to the manufacturer’s directions (Caspase-3 Colorimetric Correlated Assay Kit, Assay Designs, Inc., Ann Arbor, MI, USA).

### Immunodetection of STAT3 by Fluorescence Microscopy

Cardiomyocytes were plated on collagen pre-treated glass coverslips. After treatments, myocytes were washed twice with PBS and fixed for 40 minutes with 4% paraformaldehyde. After permeabilization with 0.2% Triton-X100 in PBS, cells were blocked with 1% of fatty acid-free albumin and incubated overnight with primary rabbit anti-STAT3 antibodies at 4°C. The next day, myocytes were washed with PBS and incubated with Alexa 488-conjugated goat anti-rabbit antibody for 2 hours. Myocytes were then mounted and visualized in an Olympus AX70 Research Microscope.

### Statistical Analysis

Data are presented as means ± SEM. Statistical differences were determined using one-way ANOVA followed by Tukey’s multiple comparison post-hoc test or an unpaired two-tailed Student’s t-test. Differences between treatment groups were considered significant at a level of *P*<0.05.

## Results

### Leptin-induces mPTP Opening

Our first set of studies was aimed at determining whether leptin can directly induce mPTP opening. As demonstrated in Figure 1 (panels A and B) leptin significantly decreased calcein-AM fluorescence by approximately 20% indicative of increased mPTP opening. However, in digitonin-permeabilized myocytes in the presence of calcium, the ability of leptin to increase mPTP opening was markedly accentuated to nearly 40% (Figure 1C and 1D). In both intact myocytes as well as digitonin-permeabilized myocytes, the effect of leptin was prevented by the mPTP inhibitor sanglifehrin A (SfA). We also compared the effect of leptin with the mPTP opener atractyloside. As shown in Figure 1C, atractyloside produced a robust mPTP opening of approximately 60%, an effect which was completely abrogated by SfA (not shown). No effect of leptin was observed when the treatment period was reduced to 4 hours (Figure 1E).

### Studies into Potential Role of STAT Activation

Many of the effects of leptin are related to its ability to activate the Janus kinase/signal transducer and activator of transcription (JAK/STAT3) pathway. Accordingly, we next determined the effect of a Stat3 inhibitor peptide (SIP) on leptin-induced effects. As shown in Figure 2A leptin progressively increased Stat3 phosphorylation to values which were significantly different from control 6 and 24 h after leptin addition. Figure 2B shows complete inhibition of the leptin-induced increase in Stat3 phosphorylation by SIP whereas panels C and D show that inhibition of Stat3 phosphorylation by SIP was accompanied by a complete abrogation of leptin-induced translocation of Stat3 into nuclei.

We next determined whether leptin treatment is also associated with activation of Stat1. As shown in Figure 3A, leptin produced an early (5 and 15 minutes) and transient increase in Stat1 phosphorylation which was completely prevented by the Stat1 inhibitor fludarabine (Figure 3B) although fludarabine had no significant effect on the leptin-induced increase in Stat3 phosphorylation (Figure 3C). In contrast, SIP was effective in completely preventing the leptin-induced increase in Stat1 phosphorylation suggesting that SIP can inhibit both Stat3 as well as Stat1 phosphorylation. Although our results suggest that fludarabine is more selective as a Stat1 inhibitor compared to Stat3, this agent produced no effect on the increased mPTP opening seen following leptin addition (Figure 3E) in contrast to the effect seen with SIP.

### Relative Effects of Stat3 and ROCK Inhibition on Leptin-induced Hypertrophy and Increased mPTP Opening

As leptin has also been demonstrated to be a potent activator of the RhoA/ROCK pathway which we have shown mediates the pro-hypertrophic effect of the peptide, we also determined whether the ROCK inhibitor Y27632 could alter the ability of leptin to sensitize mitochondria to calcium as well as to compare the effect of SIP and Y27632 on the hypertrophic effect of leptin. These results are shown in Figure 4 (panels A–D) and clearly illustrate that both SIP and Y27632 were equally effective in preventing leptin-induced hypertrophy as determined by myocyte surface area and α-skeletal actin expression. However, Y27632 had no effect on increased mPTP opening in leptin-treated permeabilized myocytes (Figure 4E).

### Assessment of Apoptosis

**Figure 5:**
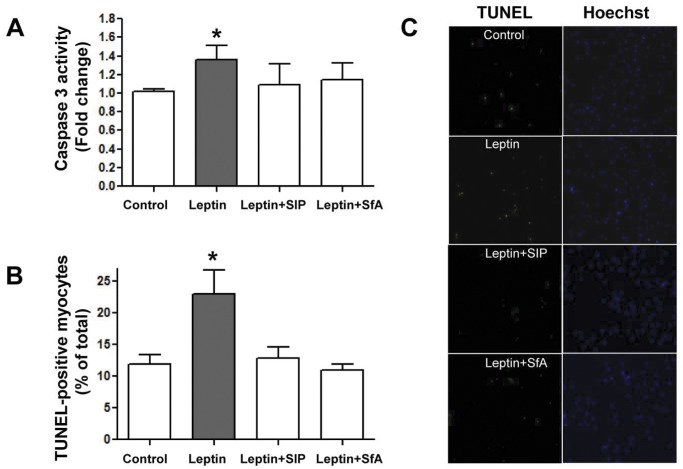
Leptin-enhances apoptosis in permeabilized myocytes. Panels A and B show that leptin significantly increases caspase 3 activity and TUNEL staining, respectively, although not in the presence of either the Stat inhibitor peptide (SIP) or sanglifehrin A (SfA). Panel C shows examples of TUNEL staining with various treatments. Values indicate mean + SEM, N = 5. *P<0.05 from control.

**Figure 6:**
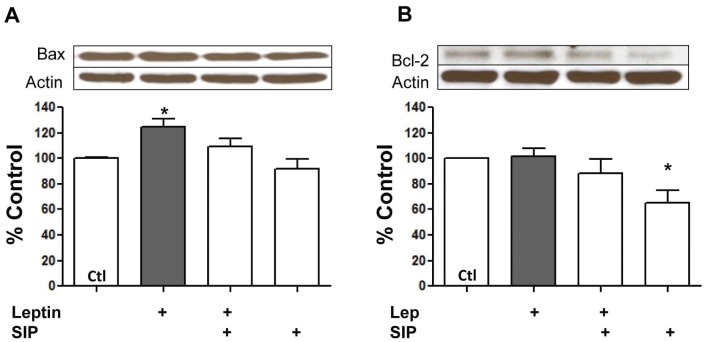
Leptin induced Bax over-expression is prevented by STAT3 inhibition. Panels show representative Western blots for Bax (panel A) and Bcl-2 (panel B) and corresponding quantified data in myocytes treated with leptin for 24 hours in the absence or presence of the Stat inhibitory peptide (SIP). Values indicate mean + SEM, N = 8. *P<0.05 from control (Ctl).

Among the consequences of increased mPTP opening is the development of apoptosis. We determined apoptosis with TUNEL staining as well as by assessing caspase 3 activity and expression of apoptosis-related proteins. These results are summarized in Figure 5 which show that leptin exerted increased caspase 3 activity by approximately 30% (<P<0.05, Panel A) in myocytes and significantly increased the number of TUNEL-positive myocytes by nearly two-fold (panels B and C). The effect of leptin was prevented by the SIP as well as by the inhibitor of mPTP opening SfA. Similarly, we also determined protein expression levels of the pro- and anti-apoptotic factors Bax and Bcl-2, respectively. As summarized in Figure 6, leptin significantly increased protein expression of Bax (panel A) whereas Bcl-2 protein levels were unaffected (panel B). This figure also demonstrates a direct effect of the SIP on Bcl-2 protein levels as evidenced by a significant reduction in the absence of any treatment, although Bax levels were unaffected.

## Discussion

The goals of the present study were to determine whether leptin can affect mitochondrial function especially related to mPTP opening and/or whether it can alter the mitochondrial response to calcium overload in digitonin-permeabilized myocytes. Elevated levels of leptin have been associated with increased cardiovascular risk particularly with respect to heart failure [2]–[5] and hyperleptinemia has been proposed as a potential marker for heart failure development [26]. The role of leptin in the heart failure processes is still somewhat uncertain as both deleterious [6], [10]–[16] and beneficial [17], [27] roles have been proposed. The bases for such varied reported effects of leptin are unknown but may reflect different experimental models or leptin concentrations among other factors [18]. Clinical studies have revealed hyperleptinemia in patients with heart failure, independently of obesity, and various studies have suggested leptin as a contributing factor to the development of heart failure [2]–[5]. Interestingly, a recent study has proposed leptin as a contributing factor for developing heart failure in obese men with no history of preexisting coronary heart disease but not in heart failure patients with coronary heart disease [4]. Although further studies are necessary, based on this finding it has been postulated that leptin may directly contribute to the development of heart failure in obese individuals [28].

**Figure 7:**
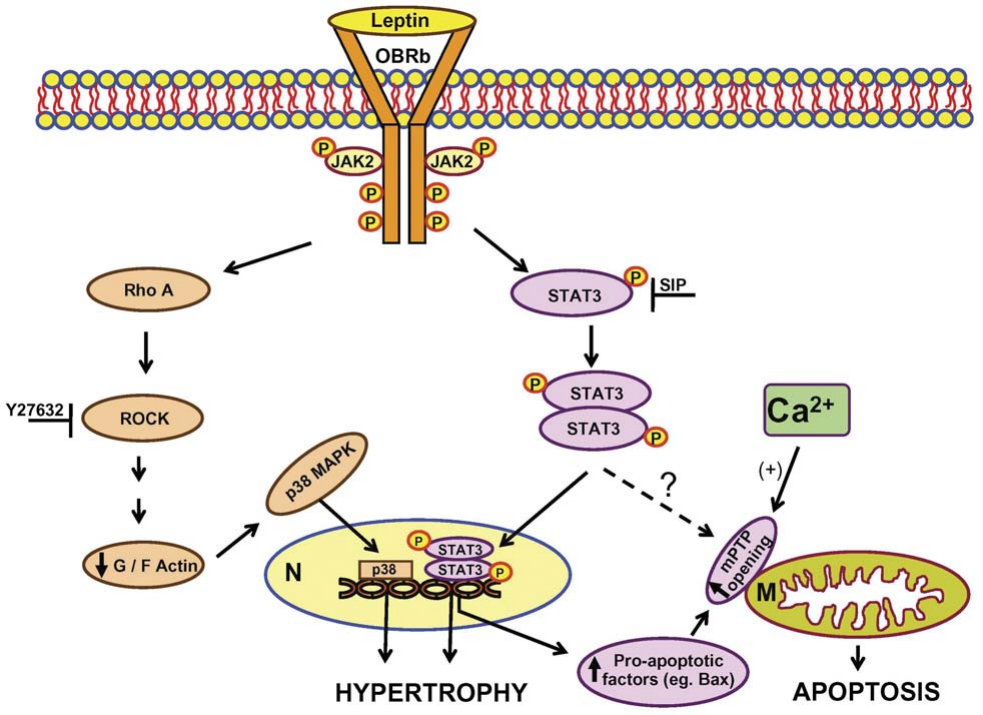
Schematic illustrating potential mechanisms underlying the pro-hypertrophic and pro-apoptotic effects of leptin in cardiomyocytes. Please refer to Discussion for details.

In recent years mitochondria have emerged as important players in the evolution to heart failure. The primary function of mitochondria under physiological conditions is the generation of adenosine triphosphate through oxidative phosphorylation [29]. Thus, these organelles, which occupy more than 30% of cardiomyocyte volume, are critical for cell survival. However, abnormal elevations in intracellular calcium concentrations induce the formation of mPTPs in the inner mitochondrial membrane [30]. As recently reviewed, the consequences of increased mPTP opening are substantial and among these responses is the release of pro-apoptotic proteins due to rupture of the outer mitochondrial membrane [31], [32].

Our results indicate that 24 hour exposure to leptin, at a concentration (50 ng/ml, 3.1 nM) well within plasma levels reported in obese individuals [1], has a modest but significant direct effect on mPTP opening in normal cardiomyocytes which does not translate into pathological responses. However, our results also show that leptin markedly sensitizes mitochondria to the detrimental effects of calcium in permeabilized myocytes resulting in substantial mPTP opening, although less than that seen with the mPTP activator atractyloside. This effect was translated to significantly increased markers of apoptosis as evidenced by increased TUNEL staining, caspase 3 activity and Bax protein expression.

The cardiac effects of leptin are likely mediated by multiple cell signalling process [33]. However, the long form of the leptin receptor family (termed LepRb or OBRb) is a type of class 1 cytokine receptor linked to Stat3 activation [34]. Although there is substantial evidence that Stat3 activation is favorable process resulting in diminished myocardial remodelling and apoptosis [35] other studies have shown that Stat3 mediates the pro-hypertrophic effects of various factors including leptin as well as α_1_ adrenoceptor activation [11], [36]. Moreover, Stat3 inhibition regresses myocardial remodelling and hypertrophy in a rat *in vivo* model produced by renal artery constriction [37]. The present study suggests that Stat3 is also a critical mediator of leptin-induced mitochondrial changes since leptin administration resulted in increased Stat3 phosphorylation as well as its translocation into nuclei. In addition, a Stat3 inhibitor peptide completely prevented leptin-induced mPTP opening and development of apoptosis as well as a complete abrogation of the hypertrophic response to leptin. We considered the possibility that the effect of SIP was potentially due to inhibition of Stat1. Indeed, leptin-induced Stat1 activation was also inhibited by SIP although Stat1 activation was of a transient nature occurring early after leptin administration and reversing to control values after 60 minutes. Moreover, the selective Stat1 inhibitor fludarabine, which completely suppressed Stat1 activation failed to alter leptin-induced mPTP opening. Taken together, our results provide evidence for a Stat3-dependent leptin induced mPTP opening which contributes to increased apoptosis in permeabilized ventricular myocytes. Although this study contrasts reports of anti-apoptotic effects of Stat3 activation as well as its ability to inhibit mPTP opening [38], [39] this may reflect the nature or duration of the stimulus. With respect to the latter, it is possible that in short-term insult, such as acute myocardial ischemia and reperfusion, Stat3 may contribute to mitochondrial and cardiac protection [40], [41], in contrast to 24 hour stimulation as carried out in the present study. Indeed, this difference in treatment duration may be particularly critical and of importance with respect to leptin as the peptide has been shown to exert a cardioprotective influence against acute myocardial ischemia (35 min) and reperfusion (120 min) [40]. Another possible contribution to the apparent discrepancy may reflect the nature of the stimulus and whether co-processes such as activation of other pro-apoptotic factors contribute to the overall role of Stat3 under pathological conditions. An interesting example of this was suggested in a recent study showing that human donor heart dysfunction prior to potential transplantation is associated with markedly increased Stat3 activation as well as increased gene expression of the pro-apoptotic mitochondrial protein BNIP3, possibly secondary to increased Stat3-dependent nitric oxide (NO) production [42]. Although leptin has been shown to increase NO production [43], whether this mediates the pro-apoptotic effect of leptin in the present investigation and whether this is dependent on BNIP3 upregulation is not currently known but deserving of future studies.

We have previously documented a potent ability of leptin to activate the RhoA/ROCK pathway and demonstrated a critical importance of the latter in mediating the pro-hypertrophic effects of leptin in cardiomyocytes which was dependent on increased actin polymerization [12]. Therefore, to further obtain potential mechanistic insights into the mitochondrial effects of leptin we determined the possible contribution of the RhoA/ROCK pathway by assessing the effect of the ROCK inhibitor Y27632. However, Y27632 was ineffective in preventing leptin-induced mPTP opening at a concentration which completely suppressed the hypertrophic response to the peptide. This finding suggests that RhoA/ROCK activity in not involved in leptin-induced mPTP opening in the present model. Moreover, the finding indicates a dissociation between the pro-hypertrophic effects of leptin and its ability to sensitize mitochondria to mPTP opening indicating that increased mPTP opening can occur in the absence of hypertrophy as evidenced in Y27632-treated myocytes.

A schematic of our working hypothesis regarding both the hypertrophic and pro-apoptotic effects of leptin is shown in Figure 7. Activation of the long form of the leptin receptor (OBRb) leads to stimulation of the RhoA/ROCK pathway resulting in increased actin polymerization as reflected by a reduced G to F actin ratio which further results in increased p38 MAPK translocation into nuclei and the resultant hypertrophic response [44]. Based on results reported here, concomitant activation of Stat3 also contributes to the hypertrophic response by virtue of its ability, in its phosphorylated dimeric form, to enter nuclei resulting in increased transcription. Furthermore, we propose that this also results in *de novo* synthesis of various pro-apoptotic factors. Among these is Bax which has recently been shown to be an important factor in reducing the threshold for mPTP opening [45] thereby producing mitochondrial changes by increasing mPTP opening, an effect facilitated by elevations in intracellular Ca^2+^ concentrations. As Stat3 can also associate with mitochondria where it is known to regulate mitochondrial respiration [46] it is also possible that the effects of leptin reported here reflect a direct mitochondrial effect of Stat3, although this issue requires in-depth further study.

A limitation of our study is the use of neonatal myocytes, however this was necessitated by the fact that 24 hour treatment with leptin was required to observe mitochondrial remodelling with no responses seen after 4 hours of leptin treatment. Adult cardiac myocytes are very difficult to maintain in culture and undergo substantial morphological changes after 24 hour culture conditions. Accordingly, the relevance of our findings to the clinical scenario must be done cautiously but it is important to note that obesity in both experimental animals as well as in the human population is associated with mitochondrial dysfunction and activation of pro-apoptotic pathways [47], [48]. Our results warrant further studies to determine whether hyperleptinemia associated with obesity contributes to mitochondrial-related cardiac pathology and whether this can be modified by therapeutic approaches aimed at attenuating the effects of leptin, such as the use of leptin receptor antagonists which may have therapeutic potential [49].
